# [Corrigendum] miR-504 promotes tumour growth and metastasis in human osteosarcoma by targeting TP53INP1

**DOI:** 10.3892/or.2025.8914

**Published:** 2025-05-14

**Authors:** Qingchun Cai, Sixiang Zeng, Xing Dai, Junlong Wu, Wei Ma

Oncol Rep 38: 2993–3000, 2017; DOI: 10.3892/or.2017.5983

Following the publication of the above article, an interested reader drew to the authors' attention that, concerning the Transwell assay experiments shown in Fig. 2F on p. 2997, the ‘Blank’ and ‘Normal control’ data panels featured a strikingly similar overlapping area, such that these data had apparently been derived from the same original source where the results of differently performed experiments were meant to have been shown.

After having re-examined their original data, the authors realized that Fig. 2F had inadvertently been assembled incorrectly; however, the authors were able to repeat these experiments, and the revised version of Fig. 2, now including new data for the experiments shown in Fig. 2F, is shown on the next page. Note that the errors made in terms of assembling the data in Fig. 2 did not greatly affect either the results or the conclusions reported in this paper, and all the authors agree to the publication of this corrigendum. The authors regret that these errors went unnoticed prior to the publication of their article, and are grateful to the Editor of *Oncology Reports* for allowing them this opportunity to publish this corrigendum. They also apologize to the readership for any inconvenience caused.

## Figures and Tables

**Figure 2.**
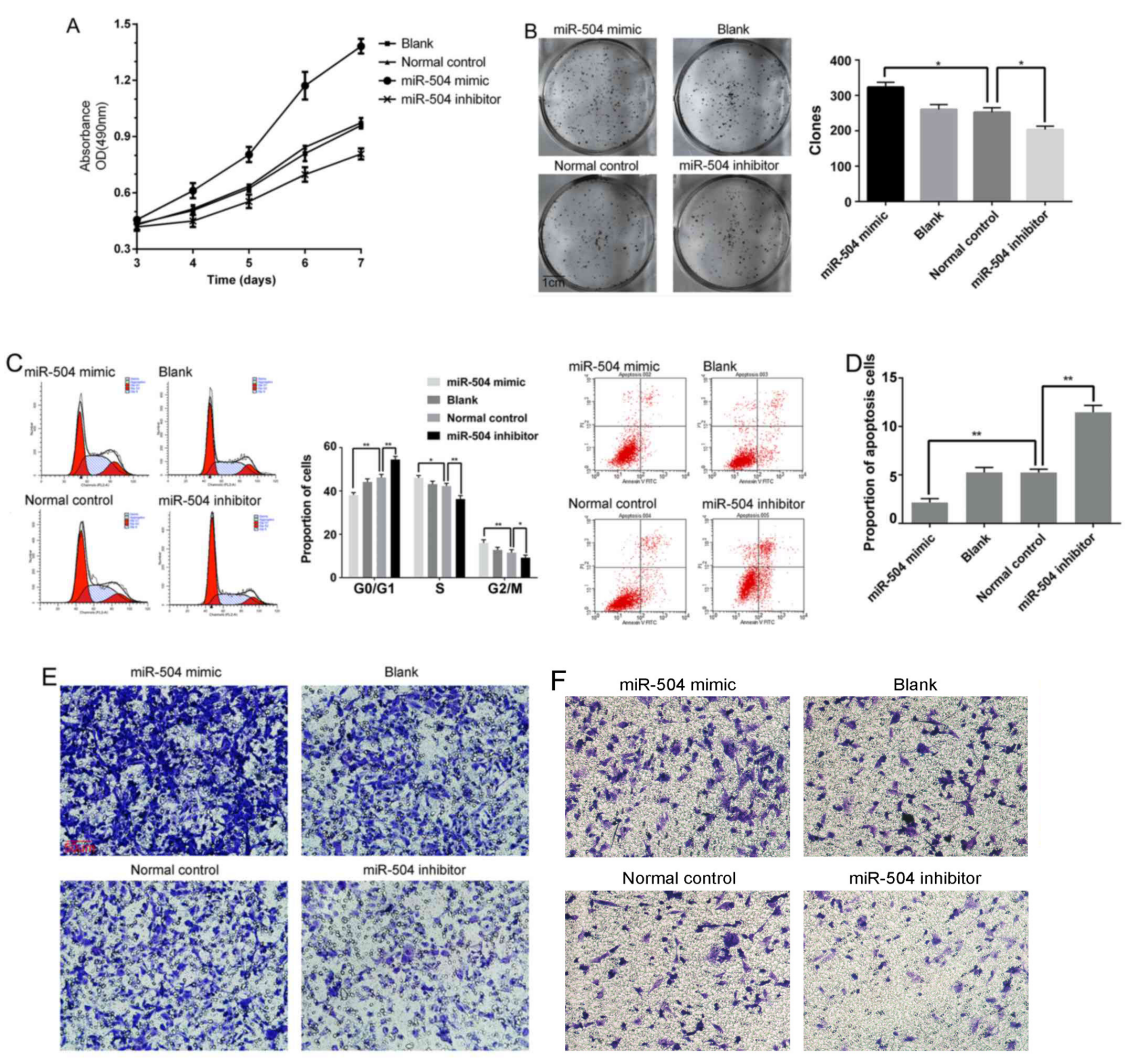
miR-504 enhances 143B cell growth and promotes metastasis *in vitro* by targeting TP53INP1. (A) The Cell Counting Kit-8 assay was performed to evaluate cell proliferation 3–7 days post-transfection. (B) The clone formation assay was performed to examine cell activity at 9 days post-transfection. (C) The cell cycle distribution was determined by flow cytometric analysis with propidium iodide staining. (D) The proportion of apoptotic cells was determined by flow cytometric analysis with Annexin-V and propidium iodide staining. (E) Transwell assay results. The images show the cells that travelled through the micropore membrane without Matrigel. (F) Transwell assay results. The images show the cells that travelled through the micropore membrane with Matrigel. Normal control, 143B cells transfected with the normal control; miR-504 mimic, 143B cells transfected with the miR-504 mimic; miR-504 inhibitor, 143B cells transfected with the miR-504 inhibitor. *P<0.05, **P<0.01 vs. the normal control. All experiments were performed in triplicate. TP53INP1, tumour protein p53-inducible nuclear protein 1.

